# *In utero* Undernutrition Programs Skeletal and Cardiac Muscle Metabolism

**DOI:** 10.3389/fphys.2015.00401

**Published:** 2016-01-06

**Authors:** Brittany Beauchamp, Mary-Ellen Harper

**Affiliations:** Department of Biochemistry, Microbiology, and Immunology, Faculty of Medicine, University of OttawaOttawa, ON, Canada

**Keywords:** intrauterine growth restriction, metabolic programming, mitochondria, oxidative phosphorylation, uncoupling, epigenetics

## Abstract

*In utero* undernutrition is associated with increased risk for insulin resistance, obesity, and cardiovascular disease during adult life. A common phenotype associated with low birth weight is reduced skeletal muscle mass. Given the central role of skeletal muscle in whole body metabolism, alterations in its mass as well as its metabolic characteristics may contribute to disease risk. This review highlights the metabolic alterations in cardiac and skeletal muscle associated with *in utero* undernutrition and low birth weight. These tissues have high metabolic demands and are known to be sites of major metabolic dysfunction in obesity, type 2 diabetes, and cardiovascular disease. Recent research demonstrates that mitochondrial energetics are decreased in skeletal and cardiac muscles of adult offspring from undernourished mothers. These effects apparently lead to the development of a thrifty phenotype, which may represent overall a compensatory mechanism programmed *in utero* to handle times of limited nutrient availability. However, in an environment characterized by food abundance, the effects are maladaptive and increase adulthood risks of metabolic disease.

Early life environmental factors, such as maternal food restriction, contribute to the development of metabolic diseases in offspring (Gluckman et al., 2008). Intrauterine growth restriction (IUGR) is one environmental perturbation that has been linked to the development of obesity and type 2 diabetes mellitus (T2DM). The idea that prenatal events may be important in determining risk for adult disease was first reported by David Barker who made a landmark observation that birth weight is inversely correlated with the risk of coronary heart disease in adulthood (Barker et al., 1989). The birth records of 16,000 men and women who were born in Hertfordshire between 1911 and 1930 were examined. Death from coronary heart disease was associated with low birth weight, with the rates falling progressively between individuals with a birth weight less than 2500 g and individuals with a birth weight of 4310 g.

Low birth weight is defined by the World Health Organization as weight at birth < 2500 g (World Health Organization United Nations Children's Fund, 2004). 15.5% of all babies are born with low birth weight, representing over 20 million infants worldwide (World Health Organization United Nations Children's Fund, 2004). While the incidence of low birth weight is greater in developing countries, it remains a significant problem in developed countries as well. In North America, 7.7% of infants are low birth weight (World Health Organization United Nations Children's Fund, 2004). Low birth weight may be a result of preterm birth or poor fetal growth. Poor fetal substrate supply can be due to poor maternal energy intake (insufficient intake of a specific micro- or macronutrient, or reduced total calories), placental insufficiency, maternal smoking, pregnancy at high altitude, or high maternal levels of stress hormones (e.g., cortisol). Interestingly, infants who are born with a high birth weight are also susceptible to metabolic disease (Boney et al., 2005). Studies have shown that there is a U-shaped correlation between birth weight and obesity with a higher prevalence of obesity for low birth weight and high birth weight (McCance et al., 1994; Wei et al., 2003). Here the focus will be on low birth weight as a result of poor fetal substrate supply and its impact on skeletal and cardiac muscle. These tissues have high metabolic demands and are known to be sites of major metabolic dysfunction in chronic diseases such as T2DM and cardiovascular disease.

After Barker's initial observation, subsequent epidemiological studies showed a strong correlation between *in utero* undernutrition, low birth weight, and risk of adult cardiovascular disease, impaired glucose tolerance, T2DM, and obesity (Figure 1) (Hales et al., 1991; Barker et al., 1993; Ravelli et al., 1999; Roseboom et al., 2000; Painter et al., 2005). The well-studied epidemiological data from the Dutch Hunger Winter show the importance of adequate fetal nutrition. During this short-term famine in 1944–1945, the daily nutritional intake was reduced to ~400–1000 kcal. Adults whose mothers were exposed to the famine during pregnancy had low birth weight and had impaired glucose tolerance and predisposition to T2DM (Hales et al., 1991). These studies gave rise to the “developmental origins of adult disease” hypothesis, which states that adverse influences early in development result in physiological adaptations that increase susceptibility to adult disease. The increased risk of obesity, insulin resistance, and T2DM has been suggested to be due to a thrifty phenotype programmed *in utero* that endows offspring with an increased capacity to store fuels rather than burning them (Hales and Barker, 1992). This apparent adaptive response by the fetus involves metabolic alterations that could altogether conserve energy expenditure to allow growth of key organs such as the brain, at the expense of other tissues such as muscle. Thus, when the nutrients provided to a fetus are limited, the fetus adapts to this environment through physiological changes that enhance its survival under these conditions. However, if the fetus is born into an environment in which nutrients are abundant, the adaptations made *in utero* may become a disadvantage (Gluckman and Hanson, 2004). Thus, disparities between the predicted environment and the actual environment into which the child is born may result in an increased disease risk.

**Figure 1:**
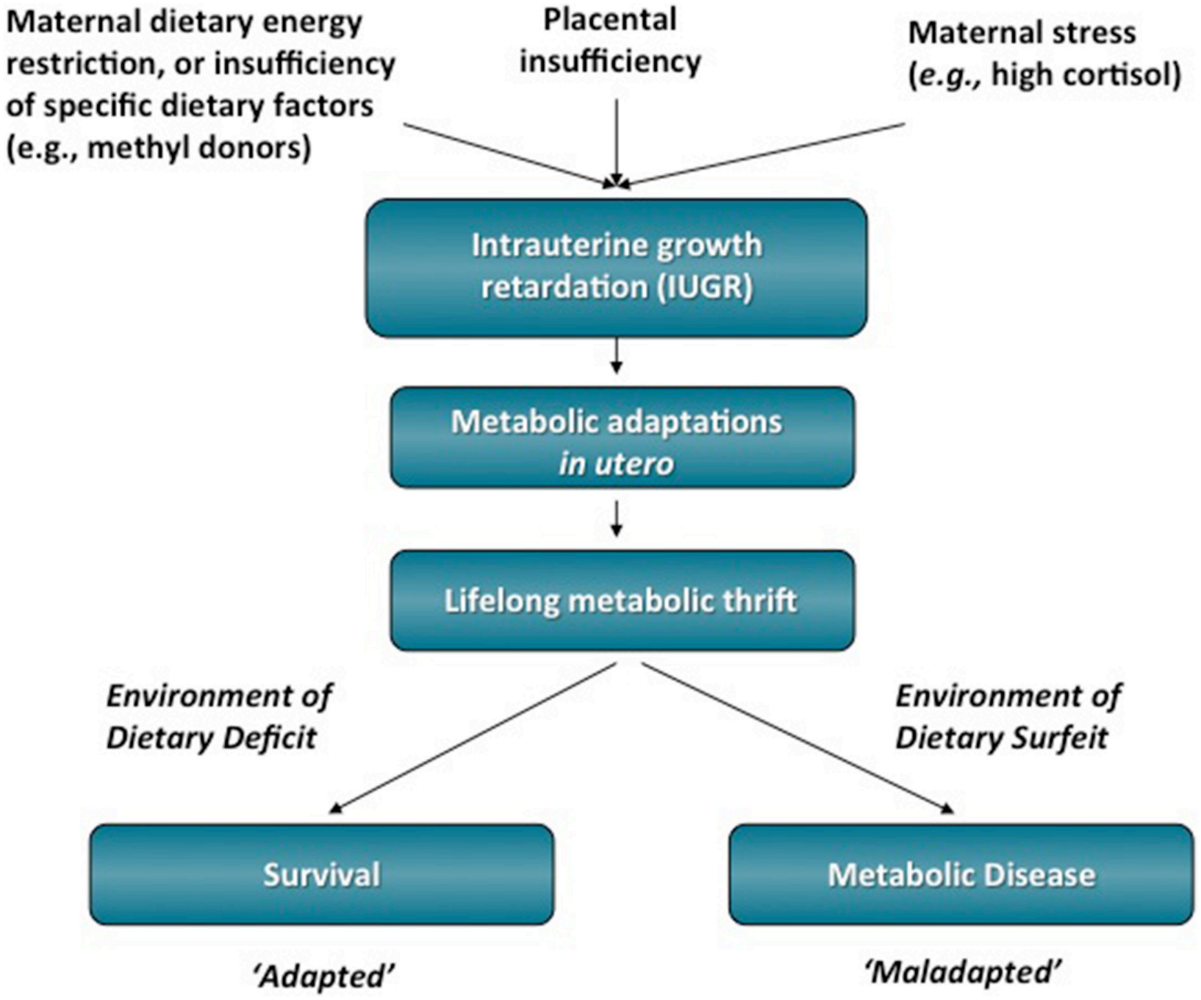
**IUGR is one environmental perturbation that has been linked to the development of T2DM and obesity in adulthood**. It is hypothesized that the early life stressor of dietary energy restriction may program metabolic adaptations that favor survival initially, but are ultimately detrimental to adult health in an environment of dietary energy surfeit. Therefore, what was an advantage *in utero* in which energy substrates was scarce can become a disadvantage by increasing the person's susceptibility to metabolic diseases in adulthood.

Research based on animal models of IUGR has provided extensive support for the findings from human epidemiological studies and has substantially advanced our understanding of the negative impact of a suboptimal *in utero* environment. The most commonly used animal models of IUGR are maternal caloric or protein restriction and induction of uteroplacental insufficiency. These models have shown that a suboptimal *in utero* environment has deleterious consequences for adult health, with effects in many organs and tissues including skeletal muscle, heart, pancreas, liver, blood, and the brain (Snoeck et al., 1990; Woodall et al., 1996a,b; Park et al., 2003, 2004; Peterside et al., 2003; Qiu et al., 2004; Jimenez-Chillaron et al., 2005, 2009; Bubb et al., 2007; Schober et al., 2009; Woo et al., 2011; Fung et al., 2012; Thorn et al., 2013; Tare et al., 2014; Beauchamp et al., 2015a,b). We are only just beginning to understand the profound impact of suboptimal *in utero* nutrition on adult metabolic health.

A common phenotype in IUGR humans and animals is reduced lean mass (Hediger et al., 1998; Jimenez-Chillaron et al., 2005; Kensara et al., 2005; Wells et al., 2007). Lean body mass, primarily skeletal muscle, is known to be the best predictor of basal metabolic rate (Zurlo et al., 1990; Rolfe and Brown, 1997). Skeletal muscle comprises ~40% of the body mass in an adult human and although its metabolic rate per gram of tissue is relatively low, it greatly contributes to metabolic rate due to its high fractional contribution to body mass (Zurlo et al., 1990; Rolfe and Brown, 1997). Therefore, differences in muscle metabolism have potentially substantial implications in determining one's susceptibility to obesity and related metabolic disease, such as T2DM. Indeed, skeletal muscle is the largest insulin-sensitive tissue in the body and is the primary site for insulin-stimulated glucose utilization (Defronzo et al., 1985). Thus, blood glucose homeostasis, particularly in the post-prandial state is greatly impacted by insulin resistance in muscle. As muscle is a key determinant of whole body metabolism and insulin sensitivity, reductions in muscle mass and/or function may be especially important to the increased metabolic disease risk (Defronzo et al., 1985; Zurlo et al., 1990; Rolfe and Brown, 1997).

In addition to reduced lean mass, low birth weight is associated with altered skeletal muscle fiber composition, and oxidative capacity (Figure 2). Human studies have documented a shift toward more type II glycolytic fibers, which accompanied skeletal muscle insulin resistance (Jensen et al., 2007). When challenged with a hyperinsulinemic-euglycemic clamp, a measure of tissue insulin sensitivity, those who had a low birth weight had decreased glucose uptake, consistent with impaired insulin sensitivity (Jaquet et al., 2000). People with low birth weight have also been shown to have reduced muscle glucose uptake after local insulin infusions and decreased expression of insulin signaling proteins and glucose transporter 4 (GLUT4) in skeletal muscle (Hermann et al., 2003; Ozanne et al., 2005; Jensen et al., 2008). In more rigorously controlled animal models of low birth weight, many of these same skeletal muscle alterations have also been observed. In IUGR animal models, skeletal muscle has reduced mass, decreased GLUT4 expression, decreased glycogen content, decreased insulin-stimulated glucose uptake, decreased oxidative capacity, and increased lipid accumulation (Selak et al., 2003; Jimenez-Chillaron et al., 2005; Zhu et al., 2006; Raychaudhuri et al., 2008; Huber et al., 2009; Dai et al., 2012; Beauchamp et al., 2015a).

**Figure 2:**
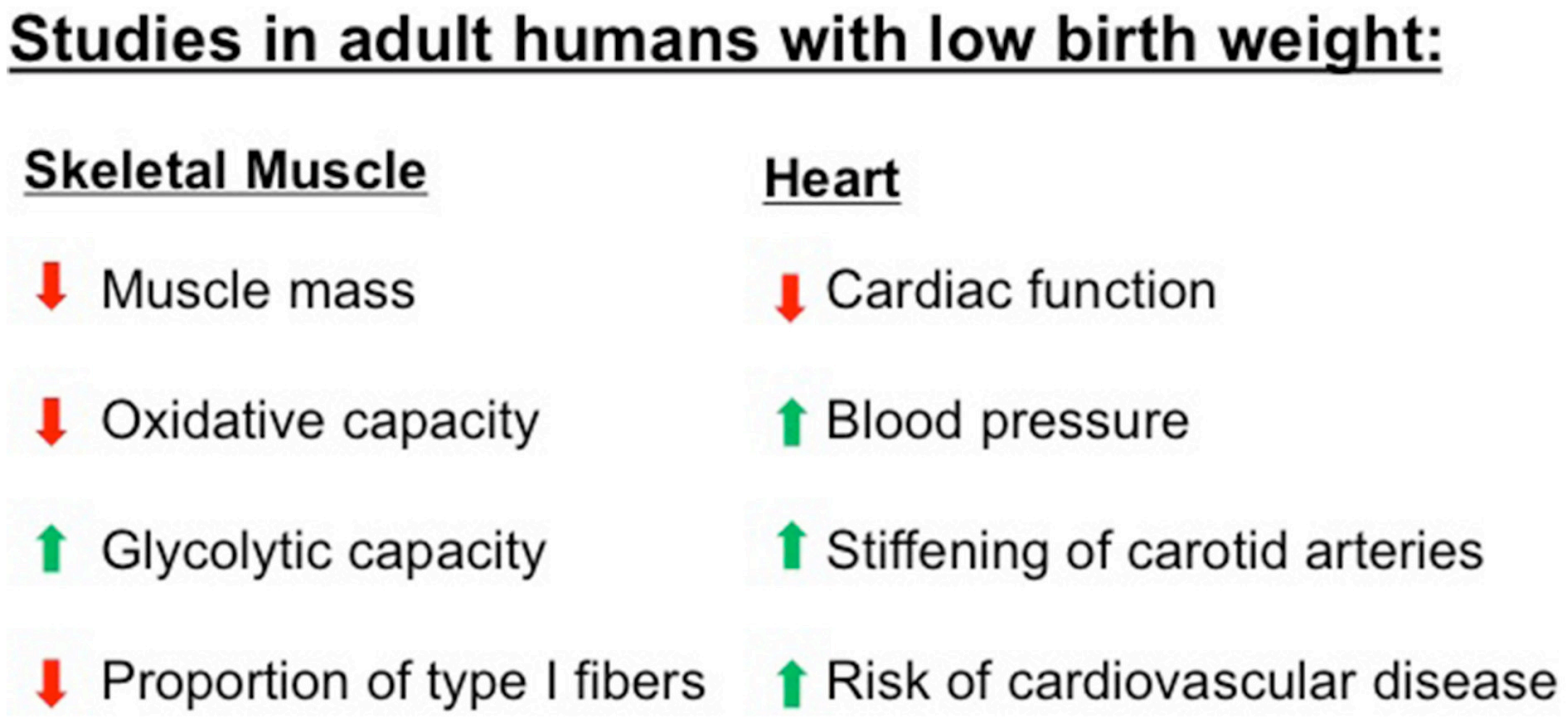
**Numerous studies have demonstrated that adult humans who were born with low birth weight have abnormalities in characteristics of skeletal muscle and heart, two tissues that have high metabolic demands**. These abnormalities increase the risk for metabolic diseases including obesity, type 2 diabetes, and cardiovascular diseases. Please refer to text for specific references.

Recently, we have used a mouse model system of maternal undernutrition during late pregnancy to examine offspring from undernourished dams. Consistent with previous studies, these low birth weight offspring had increased adiposity and decreased glucose tolerance in adulthood compared to controls (Beauchamp et al., 2015a). Our studies focused on female offspring, as our pilot studies indicated a more pronounced metabolic phenotype than in male offspring. In permeabilized fiber preparations from mixed fiber type muscle of adult females, *in utero* undernourished mice had decreased mitochondrial content and decreased mitochondrial proton leak respiration, fatty acid oxidative capacity, and state three respiratory capacity through complex I (Beauchamp et al., 2015a). The findings have implications for obesity risk. Obesity is a result of an energy imbalance, in which energy intake exceeds energy expenditure over a sustained period of time. In the long-term this results in energy storage in the form of triglycerides in adipose tissue. Therefore, our findings of decreased mitochondrial content and decreased capacity for fuel oxidation in muscle, an indicator of tissue energy expenditure, may in part explain the increased susceptibility to obesity in IUGR offspring. Furthermore, we have shown that IUGR offspring lose less weight after a 4 week 40% calorie restriction diet (Beauchamp et al., 2015a). We have suggested that this resistance to weight loss may be due to the thrifty metabolic mechanisms programmed in skeletal muscle *in utero*, and may have implications for diet-resistant obesity, which we investigate in human clinical populations (Harper et al., 2002; Gerrits et al., 2010; Thrush et al., 2014). Thus, it seems that *in utero* undernutrition not only increases susceptibility to obesity but may also make weight loss more difficult.

It has been hypothesized that mitochondrial programming may be a key adaptation made by an IUGR fetus to promote survival in a nutrient-restricted environment (Lee et al., 2005). Mitochondria play a key metabolic role and are responsible for oxidizing energy substrates to support ATP synthesis, which can then be used to drive a very wide range of energy demanding reactions in cells. Mitochondrial dysfunction is implicated in many disease states, including obesity and T2DM and thus, mitochondrial dysfunction may be a link between *in utero* nutrition and health and disease in adult life. IUGR has been associated with decreased skeletal muscle mitochondria DNA content and decreased expression levels of genes involved in mitochondrial biogenesis and function (Lane et al., 1998; Park et al., 2004; Liu et al., 2012). Consistent with these findings, we have shown that *in utero* undernourished offspring have decreased skeletal muscle mitochondrial content and impaired mitochondrial function (Beauchamp et al., 2015a). Moreover, we assessed energetics in isolated mitochondria and found that mitochondria from *in utero* undernourished offspring have decreased coupled and uncoupled respiration compared to mitochondria from control mice (Beauchamp et al., 2015a). Therefore, we have shown that not only do IUGR offspring have decreased skeletal muscle mitochondrial content but respiration per mitochondrion is also decreased. These skeletal muscle adaptations are consistent with a programmed thrifty phenotype, which would set the stage for the development of adult metabolic disease in an environment with abundant nutrition.

Given the high energy requirements of the heart, IUGR may be associated with cardiac metabolic alterations that have negative effects in adulthood. Many cardiac diseases and heart failure are associated with altered metabolism in the heart, including a general decrease in oxidative capacity and the down-regulation of enzymes of fatty acid oxidation (Sack et al., 1996; Sharov et al., 2000; Razeghi et al., 2001; Stanley et al., 2005; Boudina et al., 2007; Anderson et al., 2009). In humans, IUGR is associated with changes in cardiac morphology, premature stiffening of carotid arteries, impaired cardiac function, and elevated blood pressure (Martin et al., 2000; Bahtiyar and Copel, 2008; Crispi et al., 2010). In animal models, IUGR is associated with the development of adult hypertension, vascular dysfunction, and increased myocardial lipid content (Battista et al., 2002; Cheema et al., 2005; Zohdi et al., 2012). IUGR rats have an increased susceptibility to ischemia/reperfusion injury that is associated with a mismatch between myocardial glycolysis and glucose oxidation rates (Rueda-Clausen et al., 2011). In this study, IUGR offspring during reperfusion had decreased cardiac performance and significant increased amount of glucose that underwent glycolysis relative to the amount that was oxidized (Rueda-Clausen et al., 2011). Recently, we assessed energetics in a cardiac muscle homogenate and found that *in utero* undernourished mice in adulthood have decreased mitochondrial proton leak respiration (adenylate-free, and oligomycin-induced rates), fatty acid oxidative capacity, and maximum oxidative phosphorylation capacity (Beauchamp et al., 2015b). These findings are consistent with the decreased respiration in cardiac tissue reported in adults with obesity and T2DM and the decreased cardiac energy transduction associated with heart failure (Sharov et al., 1998, 2000; Boudina et al., 2007; Anderson et al., 2009; Doenst et al., 2010). Therefore, our results demonstrated that maternal undernutrition alters mitochondrial metabolism in the heart, which may contribute to the increased risk of cardiovascular and other metabolic diseases in the offspring. However, studies examining the metabolic effects of IUGR on cardiac muscle are very limited.

Skeletal muscle has a remarkable ability to adapt and respond to its environment and physiological challenges by changing its phenotype in terms of size, composition, and aerobic capacity, outcomes that are brought about by changes in gene expression, biochemical, and metabolic properties (Flück and Hoppeler, 2003; Luquet et al., 2003; Hénique et al., 2015). As such, skeletal muscle can modify its functional characteristics to adapt to metabolic need. The fetal adaptations to undernutrition that produce the long-term outcomes of IUGR are not fully understood. Intriguingly, some of these effects are transmissible across generations, suggesting that heritable changes in gene expression occur with *in utero* undernutrition. Experimental studies have shown intergenerational transmission of obesity and altered glucose metabolism associated with low birth weight (Benyshek et al., 2006; Harrison and Langley-Evans, 2009; Jimenez-Chillaron et al., 2009). The increased susceptibility to metabolic disease in adulthood may arise, at least in part, from epigenetic mediated alterations in gene expression. Epigenetic modification refers to modifications of DNA and chromatin that result in differential gene expression without altering the DNA sequence itself. These modifications include DNA methylation, genomic imprinting, and chromatin modifications such as post-translational modification of histones. These epigenetic modifications alter the binding of transcription factors to specific promoters and/or alter chromatin conformation, which in turn modulate gene expression. Thus, epigenetic modifications of the fetal genome based on maternal environmental cues may reset the metabolic state of the fetus to produce phenotypes in the offspring that are best suited for the predicted environment and that are maintained into adulthood. Evidence indicates that environmental factors acting during critical developmental periods can alter the epigenome. For example, in the mouse, the level of methyl donors, such as methionine, folate, and choline in the maternal diet has been shown to alter DNA methylation in the offspring (Wolff et al., 1998).

Human data that link maternal undernutrition to epigenetic changes are limited. In one study, whole blood genomic DNA was analyzed in adults who were *in utero* during the Dutch Hunger Winter, a period of famine, compared to their unexposed same-sex sibling. Adults who were *in utero* during the famine, and thus were undernourished, showed hypomethylation of the insulin-like growth factor II gene, a maternally imprinted gene that is a key factor in mammalian growth (Heijmans et al., 2008). Modifications to the methylation status of genes produce stable alterations in gene expression and represent a potential mechanism by which early life nutrition may influence susceptibility to metabolic disease in adulthood (Waterland and Jirtle, 2004). However, to date, epigenetic modification of muscle in IUGR offspring has not been described in humans.

Animal models are increasing our understanding of the mechanisms that cause the deleterious effects of IUGR. Epigenetic modifications that affect glucose metabolism have been described in IUGR pancreas, liver, and muscle. Pancreatic and duodenal homeobox 1 (Pdx-1) is a transcription factor that plays an important role in β-cell development and function. Expression of the *Pdx-1* promoter is decreased in IUGR and promotes the development of T2DM in adulthood. It has been shown that islets isolated from IUGR fetuses have decreased histone acetylation at the proximal promoter of *Pdx-1*, which is associated with decreased *Pdx-1* expression and defective glucose homeostasis (Pinney et al., 2011). In another study, maternal protein restriction in rats led to decreased methylation of genes for the glucocorticoid receptor (GR) and peroxisome proliferator-activated receptor alpha (PPARα) in the liver of the offspring after weaning (Jing-Bo et al., 2013). This was associated with greater mRNA expression of GR and PPARα, both of which are involved in glucose and lipid metabolism (Jing-Bo et al., 2013). In IUGR, skeletal muscle becomes insulin resistant and glucose uptake is reduced. It has been shown that IUGR is associated with alterations in transcription factor binding to the GLUT4 promoter, and this was associated with silencing histone modifications and reduced *glut4* gene expression (Raychaudhuri et al., 2008). In skeletal muscle, it has been shown that IUGR rats have increased methylation of peroxisome proliferator-activated receptor-γ coactivator-1α (PGC-1α), a master regulator of mitochondrial biogenesis (Xie et al., 2015). Accordingly, this was associated with a reduction in PGC-1α transcription activity, mitochondrial content, and protein level of components of the insulin signaling pathway (Xie et al., 2015). Taken together, these results support the idea that alterations in the maternal diet can induce epigenetic changes in muscle that are associated with altered gene expression.

While there is growing evidence for the role of epigenetics in metabolic programming in the development of chronic diseases, the detailed molecular mechanisms mediating the effects of *in utero* undernutrition remain unknown. In the future, epigenetic markers such as DNA methylation in blood and tissue samples may be able to serve as biomarkers to identify individuals at increased risk. Ultimately, this may allow prevention of disease by nutritional or pharmacological interventions.

In conclusion, *in utero* undernutrition is associated with skeletal and cardiac muscle alterations such as decreased mass, mitochondrial content, and metabolism. The adaptations in skeletal muscle are consistent with the idea that low birth weight offspring may develop a protective mechanism *in utero* for species survival in times when energy supply is restricted. However, in an environment characterized by the abundant availability of highly palatable food and a decreased need for physical activity, such adaptive mechanisms become detrimental, increasing the risk for metabolic diseases including obesity and T2DM.

## Author contributions

BB and MH wrote the manuscript, approved the final version, and agree to be accountable for all aspects of the work.

## Funding

The authors' research in this area is funded by the Canadian Institutes of Health Research, Institute of Nutrition, Diabetes and Metabolism (Grant MOP57810; to MEH), and BB was supported by an Alexander Graham Bell Canada Graduate Scholarship from the Natural Sciences and Engineering Research Council.

### Conflict of interest statement

The authors declare that the research was conducted in the absence of any commercial or financial relationships that could be construed as a potential conflict of interest.

## References

[B1] Anderson E. J. Kypson A. P. Rodriguez E. Anderson C. A. Lehr E. J. Neufer P. D. (2009). Substrate-specific derangements in mitochondrial metabolism and redox balance in the atrium of the type 2 diabetic human heart. J. Am. Coll. Cardiol. 54, 1891–1898. 10.1016/j.jacc.2009.07.031 19892241 PMC2800130

[B2] Bahtiyar M. O. Copel J. A. (2008). Cardiac changes in the intrauterine growth-restricted fetus. Semin. Perinatol. 32, 190–193. 10.1053/j.semperi.2008.02.010 18482620

[B3] Barker D. J. Gluckman P. D. Godfrey K. M. Harding J. E. Owens J. A. Robinson J. S. (1993). Fetal nutrition and cardiovascular disease in adult life. Lancet 341, 938–941. 10.1016/0140-6736(93)91224-A 8096277

[B4] Barker D. J. Winter P. D. Osmond C. Margetts B. Simmonds S. J. (1989). Weight in infancy and death from ischaemic heart disease. Lancet 2, 577–580. 10.1016/S0140-6736(89)90710-1 2570282

[B5] Battista M. C. Oligny L. L. St-Louis J. Brochu M. (2002). Intrauterine growth restriction in rats is associated with hypertension and renal dysfunction in adulthood. Am. J. Physiol. Endocrinol. Metab. 283, E124–E131. 10.1152/ajpendo.00004.2001 12067852

[B6] Beauchamp B. Ghosh S. Dysart M. W. Kanaan G. N. Chu A. Blais A.. (2015a). Low birth weight is associated with adiposity, impaired skeletal muscle energetics and weight loss resistance in mice. Int. J. Obes. (Lond). 39, 702–711. 10.1038/ijo.2014.120 25091727 PMC4326251

[B7] Beauchamp B. Thrush A. B. Quizi J. Antoun G. McIntosh N. Al-Dirbashi O. Y.. (2015b). Undernutrition during pregnancy in mice leads to dysfunctional cardiac muscle respiration in adult offspring. Biosci. Rep. 35:e00200. 10.1042/bsr20150007 26182362 PMC4613697

[B8] Benyshek D. C. Johnston C. S. Martin J. F. (2006). Glucose metabolism is altered in the adequately-nourished grand-offspring (f3 generation) of rats malnourished during gestation and perinatal life. Diabetologia 49, 1117–1119. 10.1007/s00125-006-0196-5 16557373

[B9] Boney C. M. Verma A. Tucker R. Vohr B. R. (2005). Metabolic syndrome in childhood: association with birth weight, maternal obesity, and gestational diabetes mellitus. Pediatrics 115, E290–E296. 10.1542/peds.2004-1808 15741354

[B10] Boudina S. Sena S. Theobald H. Sheng X. Wright J. J. Hu X. X.. (2007). Mitochondrial energetics in the heart in obesity-related diabetes: direct evidence for increased uncoupled respiration and activation of uncoupling proteins. Diabetes 56, 2457–2466. 10.2337/db07-0481 17623815

[B11] Bubb K. J. Cock M. L. Black M. J. Dodic M. Boon W. M. Parkington H. C.. (2007). Intrauterine growth restriction delays cardiomyocyte maturation and alters coronary artery function in the fetal sheep. J. Physiol. 578, 871–881. 10.1113/jphysiol.2006.121160 17124269 PMC2151351

[B12] Cheema K. K. Dent M. R. Saini H. K. Aroutiounova N. Tappia P. S. (2005). Prenatal exposure to maternal undernutrition induces adult cardiac dysfunction. Br. J. Nutr. 93, 471–477. 10.1079/BJN20041392 15946408

[B13] Crispi F. Bijnens B. Figueras F. Bartrons J. Eixarch E. Le Noble F.. (2010). Fetal growth restriction results in remodeled and less efficient hearts in children. Circulation 121, 2427–2436. 10.1161/CIRCULATIONAHA.110.937995 20497977

[B14] Dai Y. Thamotharan S. Garg M. Shin B. C. Devaskar S. U. (2012). Superimposition of postnatal calorie restriction protects the aging male intrauterine growth- restricted offspring from metabolic maladaptations. Endocrinology 153, 4216–4226. 10.1210/en.2012-1206 22807491 PMC3423608

[B15] Defronzo R. A. Gunnarsson R. Björkman O. Olsson M. Wahren J. (1985). Effects of insulin on peripheral and splanchnic glucose metabolism in noninsulin-dependent (type II) diabetes mellitus. J. Clin. Invest. 76, 149–155. 10.1172/JCI111938 3894418 PMC423730

[B16] Doenst T. Pytel G. Schrepper A. Amorim P. Färber G. Shingu Y.. (2010). Decreased rates of substrate oxidation *ex vivo* predict the onset of heart failure and contractile dysfunction in rats with pressure overload. Cardiovasc. Res. 86, 461–470. 10.1093/cvr/cvp414 20035032

[B17] Flück M. Hoppeler H. (2003). Molecular basis of skeletal muscle plasticity–from gene to form and function. Rev. Physiol. Biochem. Pharmacol. 146, 159–216. 10.1007/s10254-002-0004-7 12605307

[B18] Fung C. Ke X. Brown A. S. Yu X. McKnight R. A. Lane R. H. (2012). Uteroplacental insufficiency alters rat hippocampal cellular phenotype in conjunction with erbb receptor expression. Pediatr. Res. 72, 2–9. 10.1038/pr.2012.32 22367251 PMC3612538

[B19] Gerrits M. F. Ghosh S. Kavaslar N. Hill B. Tour A. Seifert E. L.. (2010). Distinct skeletal muscle fiber characteristics and gene expression in diet-sensitive versus diet-resistant obesity. J. Lipid Res. 51, 2394–2404. 10.1194/jlr.P005298 20332421 PMC2903798

[B20] Gluckman P. D. Hanson M. A. (2004). Developmental origins of disease paradigm: a mechanistic and evolutionary perspective. Pediatr. Res. 56, 311–317. 10.1203/01.PDR.0000135998.08025.FB 15240866

[B21] Gluckman P. D. Hanson M. A. Cooper C. Thornburg K. L. (2008). Effect of *in utero* and early-life conditions on adult health and disease. N. Engl. J. Med. 359, 61–73. 10.1056/NEJMra0708473 18596274 PMC3923653

[B22] Hales C. N. Barker D. J. (1992). Type 2 (non-insulin-dependent) diabetes mellitus: the thrifty phenotype hypothesis. Diabetologia 35, 595–601. 10.1007/BF00400248 1644236

[B23] Hales C. N. Barker D. J. Clark P. M. Cox L. J. Fall C. Osmond C.. (1991). Fetal and infant growth and impaired glucose tolerance at age 64. BMJ 303, 1019–1022. 10.1136/bmj.303.6809.1019 1954451 PMC1671766

[B24] Harper M. E. Dent R. Monemdjou S. Bézaire V. Van Wyck L. Wells G.. (2002). Decreased mitochondrial proton leak and reduced expression of uncoupling protein 3 in skeletal muscle of obese diet-resistant women. Diabetes 51, 2459–2466. 10.2337/diabetes.51.8.2459 12145158

[B25] Harrison M. Langley-Evans S. C. (2009). Intergenerational programming of impaired nephrogenesis and hypertension in rats following maternal protein restriction during pregnancy. Br. J. Nutr. 101, 1020–1030. 10.1017/S0007114508057607 18778527 PMC2665257

[B26] Hediger M. L. Overpeck M. D. Kuczmarski R. J. McGlynn A. Maurer K. R. Davis W. W. (1998). Muscularity and fatness of infants and young children born small- or large-for-gestational-age. Pediatrics 102, E60. 10.1542/peds.102.5.e60 9794990

[B27] Heijmans B. T. Tobi E. W. Stein A. D. Putter H. Blauw G. J. Susser E. S.. (2008). Persistent epigenetic differences associated with prenatal exposure to famine in humans. Proc. Natl. Acad. Sci. U.S.A. 105, 17046–17049. 10.1073/pnas.0806560105 18955703 PMC2579375

[B28] Hénique C. Mansouri A. Vavrova E. Lenoir V. Ferry A. Esnous C.. (2015). Increasing mitochondrial muscle fatty acid oxidation induces skeletal muscle remodeling toward an oxidative phenotype. FASEB J. 29, 2473–2483. 10.1096/fj.14-257717 25713059

[B29] Hermann T. S. Rask-Madsen C. Ihlemann N. Domínguez H. Jensen C. B. Storgaard H.. (2003). Normal insulin-stimulated endothelial function and impaired insulin-stimulated muscle glucose uptake in young adults with low birth weight. J. Clin. Endocrinol. Metab. 88, 1252–1257. 10.1210/jc.2002-021550 12629115

[B30] Huber K. Miles J. L. Norman A. M. Thompson N. M. Davison M. Breier B. H. (2009). Prenatally induced changes in muscle structure and metabolic function facilitate exercise-induced obesity prevention. Endocrinology 150, 4135–4144. 10.1210/en.2009-0125 19477938

[B31] Jaquet D. Gaboriau A. Czernichow P. Levy-Marchal C. (2000). Insulin resistance early in adulthood in subjects born with intrauterine growth retardation. J. Clin. Endocrinol. Metab. 85, 1401–1406. 10.1210/jc.85.4.1401 10770173

[B32] Jensen C. B. Martin-Gronert M. S. Storgaard H. Madsbad S. Vaag A. Ozanne S. E. (2008). Altered pi3-kinase/akt signalling in skeletal muscle of young men with low birth weight. PLoS ONE 3:E3738. 10.1371/journal.pone.0003738 19011679 PMC2580025

[B33] Jensen C. B. Storgaard H. Madsbad S. Richter E. A. Vaag A. A. (2007). Altered skeletal muscle fiber composition and size precede whole-body insulin resistance in young men with low birth weight. J. Clin. Endocrinol. Metab. 92, 1530–1534. 10.1210/jc.2006-2360 17284623

[B34] Jimenez-Chillaron J. C. Hernandez-Valencia M. Reamer C. Fisher S. Joszi A. Hirshman M.. (2005). Beta-cell secretory dysfunction in the pathogenesis of low birth weight-associated diabetes: a murine model. Diabetes 54, 702–711. 10.2337/diabetes.54.3.702 15734846

[B35] Jimenez-Chillaron J. C. Isganaitis E. Charalambous M. Gesta S. Pentinat-Pelegrin T. Faucette R. R.. (2009). Intergenerational transmission of glucose intolerance and obesity by *in utero* undernutrition in mice. Diabetes 58, 460–468. 10.2337/db08-0490 19017762 PMC2628621

[B36] Jing-Bo L. Ying Y. Bing Y. Xiang-Bing M. Zhi-Qing H. Guo-Quan H. (2013). Folic acid supplementation prevents the changes in hepatic promoter methylation status and gene expression in intrauterine growth-retarded piglets during early weaning period. J. Anim. Physiol. Anim. Nutr. (Berl). 97, 878–886. 10.1111/j.1439-0396.2012.01333.x 22853634

[B37] Kensara O. A. Wootton S. A. Phillips D. I. Patel M. Jackson A. A. Elia M. (2005). Fetal programming of body composition: relation between birth weight and body composition measured with dual-energy x-ray absorptiometry and anthropometric methods in older englishmen. Am. J. Clin. Nutr. 82, 980–987. 16280428 10.1093/ajcn/82.5.980

[B38] Lane R. H. Chandorkar A. K. Flozak A. S. Simmons R. A. (1998). Intrauterine growth retardation alters mitochondrial gene expression and function in fetal and juvenile rat skeletal muscle. Pediatr. Res. 43, 563–570. 10.1203/00006450-199805000-00001 9585000

[B39] Lee H. K. Park K. S. Cho Y. M. Lee Y. Y. Pak Y. K. (2005). Mitochondria-based model for fetal origin of adult disease and insulin resistance. Ann. N.Y. Acad. Sci. 1042, 1–18. 10.1196/annals.1338.001 15965040

[B40] Liu J. Chen D. Yao Y. Yu B. Mao X. He J.. (2012). Intrauterine growth retardation increases the susceptibility of pigs to high-fat diet-induced mitochondrial dysfunction in skeletal muscle. PLoS ONE 7:E34835. 10.1371/journal.pone.0034835 22523560 PMC3327708

[B41] Luquet S. Lopez-Soriano J. Holst D. Fredenrich A. Melki J. Rassoulzadegan M.. (2003). Peroxisome proliferator-activated receptor delta controls muscle development and oxidative capability. FASEB J. 17, 2299–2301. 10.1096/fj.03-0269fje 14525942

[B42] Martin H. Hu J. Gennser G. Norman M. (2000). Impaired endothelial function and increased carotid stiffness in 9-year-old children with low birthweight. Circulation 102, 2739–2744. 10.1161/01.CIR.102.22.2739 11094041

[B43] McCance D. R. Pettitt D. J. Hanson R. L. Jacobsson L. T. Knowler W. C. Bennett P. H. (1994). Birth weight and non-insulin dependent diabetes: thrifty genotype, thrifty phenotype, or surviving small baby genotype? BMJ 308, 942–945. 10.1136/bmj.308.6934.942 8173400 PMC2539758

[B44] Ozanne S. E. Jensen C. B. Tingey K. J. Storgaard H. Madsbad S. Vaag A. A. (2005). Low birthweight is associated with specific changes in muscle insulin-signalling protein expression. Diabetologia 48, 547–552. 10.1007/s00125-005-1669-7 15729577

[B45] Painter R. C. Roseboom T. J. Bleker O. P. (2005). Prenatal exposure to the dutch famine and disease in later life: an overview. Reprod. Toxicol. 20, 345–352. 10.1016/j.reprotox.2005.04.005 15893910

[B46] Park H. K. Jin C. J. Cho Y. M. Park D. J. Shin C. S. Park K. S.. (2004). Changes of mitochondrial dna content in the male offspring of protein-malnourished rats. Ann. N.Y. Acad. Sci. 1011, 205–216. 10.1196/annals.1293.021 15126298

[B47] Park K. S. Kim S. K. Kim M. S. Cho E. Y. Lee J. H. Lee K. U.. (2003). Fetal and early postnatal protein malnutrition cause long-term changes in rat liver and muscle mitochondria. J. Nutr. 133, 3085–3090. 14519789 10.1093/jn/133.10.3085

[B48] Peterside I. E. Selak M. A. Simmons R. A. (2003). Impaired oxidative phosphorylation in hepatic mitochondria in growth-retarded rats. Am. J. Physiol. Endocrinol. Metab. 285, E1258–E1266. 10.1152/ajpendo.00437.2002 14607783

[B49] Pinney S. E. Jaeckle Santos L. J. Han Y. Stoffers D. A. Simmons R. A. (2011). Exendin-4 increases histone acetylase activity and reverses epigenetic modifications that silence pdx1 in the intrauterine growth retarded rat. Diabetologia 54, 2606–2614. 10.1007/s00125-011-2250-1 21779870 PMC4461231

[B50] Qiu X. S. Huang T. T. Deng H. Y. Shen Z. Y. Ke Z. Y. Mei K. Y.. (2004). Effects of early nutrition intervention on IGF1, IGFBP3, intestinal development, and catch-up growth of intrauterine growth retardation rats. Chin. Med. Sci. J. 19, 189–192. 15506645

[B51] Ravelli A. C. Van Der Meulen J. H. Osmond C. Barker D. J. Bleker O. P. (1999). Obesity at the age of 50 y in men and women exposed to famine prenatally. Am. J. Clin. Nutr. 70, 811–816. 10539740 10.1093/ajcn/70.5.811

[B52] Raychaudhuri N. Raychaudhuri S. Thamotharan M. Devaskar S. U. (2008). Histone code modifications repress glucose transporter 4 expression in the intrauterine growth-restricted offspring. J. Biol. Chem. 283, 13611–13626. 10.1074/jbc.M800128200 18326493 PMC2376250

[B53] Razeghi P. Young M. E. Alcorn J. L. Moravec C. S. Frazier O. H. Taegtmeyer H. (2001). Metabolic gene expression in fetal and failing human heart. Circulation 104, 2923–2931. 10.1161/hc4901.100526 11739307

[B54] Rolfe D. F. Brown G. C. (1997). Cellular energy utilization and molecular origin of standard metabolic rate in mammals. Physiol. Rev. 77, 731–758. 9234964 10.1152/physrev.1997.77.3.731

[B55] Roseboom T. J. Van Der Meulen J. H. Osmond C. Barker D. J. Ravelli A. C. Schroeder-Tanka J. M.. (2000). Coronary heart disease after prenatal exposure to the dutch famine, 1944-45. Heart 84, 595–598. 10.1136/heart.84.6.595 11083734 PMC1729504

[B56] Rueda-Clausen C. F. Morton J. S. Lopaschuk G. D. Davidge S. T. (2011). Long-term effects of intrauterine growth restriction on cardiac metabolism and susceptibility to ischaemia/reperfusion. Cardiovasc. Res. 90, 285–294. 10.1093/cvr/cvq363 21097804

[B57] Sack M. N. Rader T. A. Park S. Bastin J. McCune S. A. Kelly D. P. (1996). Fatty acid oxidation enzyme gene expression is downregulated in the failing heart. Circulation 94, 2837–2842. 10.1161/01.CIR.94.11.2837 8941110

[B58] Schober M. E. McKnight R. A. Yu X. Callaway C. W. Ke X. Lane R. H. (2009). Intrauterine Growth Restriction Due To Uteroplacental Insufficiency Decreased White Matter And Altered Nmdar Subunit Composition In Juvenile Rat Hippocampi. Am. J. Physiol. Regul. Integr. Comp. Physiol. 296, R681–R692. 10.1152/ajpregu.90396.2008 19144756

[B59] Selak M. A. Storey B. T. Peterside I. Simmons R. A. (2003). Impaired oxidative phosphorylation in skeletal muscle of intrauterine growth-retarded rats. Am. J. Physiol. Endocrinol. Metab. 285, E130–E137. 10.1152/ajpendo.00322.2002 12637257

[B60] Sharov V. G. Goussev A. Lesch M. Goldstein S. Sabbah H. N. (1998). Abnormal mitochondrial function in myocardium of dogs with chronic heart failure. J. Mol. Cell. Cardiol. 30, 1757–1762. 10.1006/jmcc.1998.0739 9769231

[B61] Sharov V. G. Todor A. V. Silverman N. Goldstein S. Sabbah H. N. (2000). Abnormal mitochondrial respiration in failed human myocardium. J. Mol. Cell. Cardiol. 32, 2361–2367. 10.1006/jmcc.2000.1266 11113011

[B62] Snoeck A. Remacle C. Reusens B. Hoet J. J. (1990). Effect of a low protein diet during pregnancy on the fetal rat endocrine pancreas. Biol. Neonate 57, 107–118. 10.1159/000243170 2178691

[B63] Stanley W. C. Recchia F. A. Lopaschuk G. D. (2005). Myocardial substrate metabolism in the normal and failing heart. Physiol. Rev. 85, 1093–1129. 10.1152/physrev.00006.2004 15987803

[B64] Tare M. Parkington H. C. Wallace E. M. Sutherland A. E. Lim R. Yawno T.. (2014). Maternal melatonin administration mitigates coronary stiffness and endothelial dysfunction, and improves heart resilience to insult in growth restricted lambs. J. Physiol. 592, 2695–2709. 10.1113/jphysiol.2014.270934 24710061 PMC4080947

[B65] Thorn S. R. Brown L. D. Rozance P. J. Hay W. W. Jr. Friedman J. E. (2013). Increased hepatic glucose production in fetal sheep with intrauterine growth restriction is not suppressed by insulin. Diabetes 62, 65–73. 10.2337/db11-1727 22933111 PMC3526037

[B66] Thrush A. B. Zhang R. Chen W. Seifert E. L. Quizi J. K. McPherson R.. (2014). Lower mitochondrial proton leak and decreased glutathione redox in primary muscle cells of obese diet-resistant versus diet-sensitive humans. J. Clin. Endocrinol. Metab. 99, 4223–4230. 10.1210/jc.2014-1726 25148230

[B67] Waterland R. A. Jirtle R. L. (2004). Early nutrition, epigenetic changes at transposons and imprinted genes, and enhanced susceptibility to adult chronic diseases. Nutrition 20, 63–68. 10.1016/j.nut.2003.09.011 14698016

[B68] Wei J. N. Sung F. C. Li C. Y. Chang C. H. Lin R. S. Lin C. C.. (2003). Low birth weight and high birth weight infants are both at an increased risk to have type 2 diabetes among schoolchildren in taiwan. Diabetes Care 26, 343–348. 10.2337/diacare.26.2.343 12547860

[B69] Wells J. C. Chomtho S. Fewtrell M. S. (2007). Programming of body composition by early growth and nutrition. Proc. Nutr. Soc. 66, 423–434. 10.1017/S0029665107005691 17637095

[B70] Wolff G. L. Kodell R. L. Moore S. R. Cooney C. A. (1998). Maternal epigenetics and methyl supplements affect agouti gene expression in avy/a mice. FASEB J. 12, 949–957. 9707167

[B71] Woo M. Isganaitis E. Cerletti M. Fitzpatrick C. Wagers A. J. Jimenez-Chillaron J.. (2011). Early life nutrition modulates muscle stem cell number: implications for muscle mass and repair. Stem Cells Dev. 20, 1763–1769. 10.1089/scd.2010.0349 21247245 PMC3182031

[B72] Woodall S. M. Breier B. H. Johnston B. M. Gluckman P. D. (1996a). A model of intrauterine growth retardation caused by chronic maternal undernutrition in the rat: effects on the somatotrophic axis and postnatal growth. J. Endocrinol. 150, 231–242. 10.1677/joe.0.1500231 8869590

[B73] Woodall S. M. Johnston B. M. Breier B. H. Gluckman P. D. (1996b). Chronic maternal undernutrition in the rat leads to delayed postnatal growth and elevated blood pressure of offspring. Pediatr. Res. 40, 438–443. 10.1203/00006450-199609000-00012 8865281

[B74] World Health Organization United Nations Children's Fund (2004). Low Birthweight: Country, Regional And Global Estimates. New York, NY.

[B75] Xie X. Lin T. Zhang M. Liao L. Yuan G. Gao H. (2015). IUGR with infantile overnutrition programs an insulin-resistant phenotype through DNA methylation of peroxisome proliferator-activated receptor-gamma coactivator-1 alpha in rats. Pediatr. Res. 77, 625–632. 10.1038/pr.2015.32 25675425

[B76] Zhu M. J. Ford S. P. Means W. J. Hess B. W. Nathanielsz P. W. Du M. (2006). Maternal nutrient restriction affects properties of skeletal muscle in offspring. J. Physiol. 575, 241–250. 10.1113/jphysiol.2006.112110 16763001 PMC1819430

[B77] Zohdi V. Wood B. R. Pearson J. T. Bambery K. R. Black M. J. (2012). Evidence of altered biochemical composition in the hearts of adult intrauterine growth-restricted rats. Eur. J. Nutr. 52, 749–758. 10.1007/s00394-012-0381-x 22645107

[B78] Zurlo F. Larson K. Bogardus C. Ravussin E. (1990). Skeletal muscle metabolism is a major determinant of resting energy expenditure. J. Clin. Invest. 86, 1423–1427. 10.1172/JCI114857 2243122 PMC296885

